# Time allocation to active domains, physical activity, and health indicators in older adults: cross-sectional results from the OUTDOOR ACTIVE study

**DOI:** 10.1186/s12889-020-09708-z

**Published:** 2020-10-20

**Authors:** Imke Stalling, Birte Marie Albrecht, Friederike Doerwald, Karin Bammann

**Affiliations:** Institute for Public Health and Nursing Sciences (IPP), University of Bremen, Grazer Straße 2a, 28359 Bremen, Germany

**Keywords:** Time use, Physical activity, Older adults, SLOTH model, Accelerometer

## Abstract

**Background:**

Physical activity (PA) is one of the key determinants of healthy ageing. Research showed that time allocation plays an important role in PA. Therefore, an understanding of the time use of older adults is crucial for developing PA programs. The aim of this study was to examine the associations of time allocation and objectively measured PA, and several health indicators in older adults.

**Methods:**

In this cross-sectional study all 915 participants of the OUTDOOR ACTIVE study were included. The participants were 65 to 75 years old and resided in a subdistrict of Bremen, Germany (50.9% female). The active domains were derived from the SLOTH model (leisure activities, occupation, active transport, home-based activities). PA was objectively measured with accelerometers over seven consecutive days. Binary logistic regressions were used to test the associations of total PA and time spent in the domains with several health indicators (self-rated health, overweight, obesity, activities of daily living (ADL)).

**Results:**

Participants over the age of 70 years were significantly less physically active than those under 70 years and women were significantly more physically active than men. Regardless of age and sex, most time was spent on home-based activities (women: 118.5 ± 87.8 min/day; men: 80.2 ± 69.4 min/day). Both PA and time spent on leisure activities were associated with a lower risk of bad self-rated health (0.36; 95%-CL: 0.20, 0.65 for PA; 0.93; 95%-CL: 0.87, 0.99 for leisure activities) and less limitations in ADL. PA and active transport seemed to lower the risk of overweight (0.39; 95%-CL: 0.25, 0.62 for PA; 0.80; 95%-CL: 0.69, 0.93 for active transport) and obesity (0.36; 95%-CL: 0.21, 0.60 for PA; 0.77; 95%-CL: 0.64, 0.92 for active transport). Having an occupation was associated with a lower risk of bad self-rated health (0.60; 95%-CL: 0.40, 0.92).

**Conclusions:**

The results of this study provide insights in the time allocation to active domains and total PA of older adults, as well as the associations with health indicators. These findings have important implications for the development of PA programs and guidelines. Future research should examine the associations further in longitudinal studies.

**Supplementary information:**

**Supplementary information** accompanies this paper at 10.1186/s12889-020-09708-z.

## Background

One of the key determinants of healthy ageing is regular physical activity [1]. It is not only positively associated with increased quality of life and independent living, but also with decreased risks of numerous non-communicable diseases [2–4]. The World Health Organization recommends at least 150 min of moderate physical activity (PA) per week for adults, including leisure-time activities, active transportation, household chores, and occupational PA [2]. However, the prevalence of people meeting this recommendation declines with age [3]. In Germany, only 18.0% of the 60- to 69-year-olds and only 13.6% of the 70- to 79-year-olds are physically active for at least 150 min per week [5]. Accelerometers have become the preferred method of measuring total PA [6–8]. A different approach is to assess time allocation, which helps identifying daily routines and thus might be easier to translate into PA recommendations than focusing on total PA. Previous studies have associated time spent on health-related behaviours with various health indicators, including PA [9, 10]. Research suggest that time spent on leisure, home-based, occupational, and transport-related PA are associated with self-rated health and body mass index (BMI) [11].Only few time use studies investigating associations with health indicators have focused on older adults [9, 12, 13] and have rarely examined age differences within the group of older adults. Leisure time PA has several beneficial effects on health of older adults. A meta-analysis showed leisure time PA having the most benefits for mental health when compared to total PA and PA in other life domains [14]. Furthermore, it lowers the risk for all-cause, cardio-vascular disease-related, and cancer-related mortality, even in older adults, who did not increase leisure time PA until late adulthood [15]. Higher levels of leisure time PA earlier in life are positively associated with better physical functioning in older age [16]. Being physically active at work has positive effects on health [11, 17, 18], however these associations are not sufficiently researched for older adults. Most research on this age group includes the effects of retirement rather than occupation and the results regarding health are contrasting, as the review by van der Heide et al. showed [19]. A similar research situation exists in regards to transport-related PA. Active transport was found to be positively associated with better self-rated health, a lower risk for obesity as well as more overall healthy behaviours for adults [11, 20], not specifically older adults. Research on the associations of home-based activities and health indicators for this age group show inconsistent results, especially regarding self-reported health and overweight [12, 21, 22]. After retirement, the time that was previously spent at work needs to be newly allocated. Research showed that, compared to working adults, older retired people dedicate more of their time to household chores, passive as well as active leisure activities, and sleep. However, PA through leisure activities remains the domain the least time is spent on each day, pre- and post-retirement [23, 24]. This could be due to increasing sedentary time and passive leisure activities, such as watching TV, with higher age [25], which might go at the expense of time spent on active leisure time. One study by Espinel et al. [26] distinguished time allocated to self-reported sedentary, light, and moderate to vigorous PA (MVPA) in non-working adults over 65 years. The reported activities were categorised into the different PA-levels using METs. Most of time spent in MVPA was caused by household chores, with women devoting more time to them than men. Men spent more time doing leisure activities. Only 25.0% of participants met sufficient MVPA-levels through leisure activities. The study did not find any associations with age or the socioeconomic status [26]. Eibich [27], contrastingly, found that time spent on leisure activities, active and inactive, increases by 1 h per day after retirement. He also found that time spent on repairs and gardening, as well as household chores increases and concludes that most retirees invest their new found time in an active lifestyle [27]. Taking together the literature, the existing evidence suggests that most older adults invest more time into household chores as well as leisure activities and has indicated some gender differences. However, past studies on time use of older adults have often assessed PA with self-report measures. Moreover, apart from gender, little is known about how other demographic variables might affect time use. One important variable in this context might be occupational status past retirement age. Past research has shown that retirees may have more leisure time than those still working, and might invest that time in health promoting behaviours, such as physical activity [28]. In addition, there might be age differences within the group of older adults regarding time use, though this has been insufficiently studied.

Since research on time allocation and its relation with health indicators regarding older adults is scarce, the aim of this cross-sectional study is to examine the associations between time spent in active life domains, objectively measured PA, and health indicators in older adults in Germany. Specifically, we focus on the differences between two age groups (under and over 70 years) and sex. We furthermore explore differences in time allocation between working and non-working older adults. In this context, active domains comprise active leisure activities (e.g. sports, recreational walking, playing with children), occupation, active transport via bike or on foot and home-based activities (e.g. household chores, gardening).

Knowledge about time allocation of older adults to active domains and their associations with health indicators provides important background information for the development of future PA programs and guidelines.

## Methods

### Study design and population

The OUTDOOR ACTIVE study is part of the regional prevention network AEQUIPA (Physical activity and health equity: primary prevention for healthy ageing) [29]. Research goals of OUTDOOR ACTIVE are to assess prevalence of PA in older adults, explore barriers and drivers for being physically active, and to develop and implement a community-based outdoor PA promotion program [30]. First baseline data were collected between October 2015 and August 2016 using 1) a self-administered paper-pencil questionnaire regarding intrapersonal, interpersonal, and environmental determinants of PA, 2) a short physical examination (anthropometry and blood pressure) followed by a fitness test (modified public domain Senior Fitness Test [31] and handgrip strength test), and 3) accelerometry to objectively measure PA over the course of seven consecutive days. Eligibility criteria included being between 65 and 75 years old, being non-institutionalised, and living in the district Hemelingen in the city of Bremen, in the North-West of Germany. Address data were provided by the registry office in Bremen in August 2015. All eligible individuals initially received a letter and were later contacted by phone, in cases where the number could be obtained through one of the available registers. In total, 4304 individuals were registered in the study region. Of these, 615 people were excluded because of acute health problems (*n* = 242), language barriers (*n* = 22), moving out of the study region (*n* = 295), or death (*n* = 56). Out of the remaining eligible 3689 individuals, 720 were never reached, 2052 refused participation, and 915 individuals took part in the OUTDOOR ACTIVE study (response rate: 24.8%). All participants provided written informed consent and the study was approved by the ethics committee of the University of Bremen in September 2015. In the present study, all 915 OUTDOOR ACTIVE participants were included. For analyses with accelerometer data, 570 participants, who wore the accelerometer for at least 20 h a day, were included. The subjective and objective PA measurements do not assess the same kind of PA and should not be used interchangeably. We decided to use both because they can complement each other and offer a richness of information [32, 33]. Therefore, we can analyse the associations of health indicators with domain-specific PA as well as objective total PA, and get broader insights into the topic.

### Measures

#### Accelerometer-assessed physical activity

To measure PA objectively, ActiGraph GT3x-BTw accelerometers were handed to the participants following the fitness test. They were asked to wear them on seven consecutive days, ideally for 24 h straight, on their non-dominant wrist. The epoch length was set to 30 Hz. The participants were given short instructions on when to take them off (e.g. when using the sauna) and how to put them back on. Members of the project group collected the accelerometers after 1 week at the participants’ homes, cleaned them, and downloaded the data with ActiLife *(*Version 6.13.3 ActiGraph LLC, Pensacola, FL, USA). After preparing the data in ActiLife for statistical analyses, it was then transferred to the SPSS database*.* Average daily counts per minute (vector magnitudes) were included in the analyses as a continuous variable. PA was not further classified, because of the lack of established cut-offs for older adults [34] and to avoid loss of information [35]. Non-wear time was defined as 90 consecutive minutes with zero counts [36].

#### Assessment of the SLOTH model domains

We employed the time budget SLOTH model, which groups the time spent into the five domains sleep, leisure, occupation, transport, and home-based activities [37]. For this study, we only investigated active activities (see Table 1). Active activities and time spent in these domains were assessed by self-administered questionnaire that was part of the baseline survey. The questions referred to current activities but included no specific time frame. Sleep was considered a passive activity and was therefore not included in the analyses.

**Table 1:** SLOTH domains and their passive and active activities

Domains	Type	Activities
Sleep	Passive	Sleep
	Active	–
Leisure	Passive	Watching TV, reading etc.
	Active	Sports, active recreational activities, playing with children etc.
Occupation	Passive	–
	Active	All paid and unpaid work including volunteer work
Transport	Passive	Public transport, car, motor bike etc.
	Active	Cycling, walking
Home-based	Passive	–
	Active	Household chores, gardening, repairs around the house

For assessment of active leisure, we asked participants to report all currently performed organised (e.g. sports club, sports group, or a gym) as well as non-organised activities. The reported hours per week for the individual activities were added up, excluding riding a bike, since this activity could also be interpreted as a mode of transport. All paid and unpaid work including volunteer work was included in the occupation domain. Time spent on carrying out the occupation was not asked and could therefore not be included in the analyses. Time spent in active transport was assessed with a question based on the public domain Neighbourhood Environment and Walkability Scale (NEWS) [38] using 12 common destinations. The usual mode of transport as well as the minutes spent on the trips were assessed. To calculate the average daily time spent in active transport, the frequency for each destination was estimated (see additional file 1). For the analyses only transport via bike and on foot were used. Home-based activities, which comprise housework and gardening, were assessed with the same question as leisure activities (i.e., hours per week engaging in the activities). To reduce the number of outliers, the maximum possible time for housework and gardening was set to 40 h per week and for the rest of the active SLOTH dimensions to 20 h per week.

#### Assessment of health indicators

Self-rated health and activities of daily living (ADL) were assessed via questions from the public domain SF-36 v1.0 questionnaire [39, 40]. BMI was calculated using body weight (in kg) and height (in m), both measured during the short physical examination with a Kern MPC 250 K 100 M personal floor scale (Kern & Sohn GmbH, Ballingen, Germany) and a Seca 217 mobile Stadiometer (Seca GmbH & Co. KG, Hamburg, Germany), respectively. The classification by the WHO [41] was used for the distinction between normal weight, overweight, and obesity.

#### Sociodemographic information

Participant’s age, sex, marital status, educational status, net household income, and occupational status were assessed by a self-administered questionnaire. To assign each participant a socio-economic status (SES), an additive social class index was calculated using educational years (school years and training years combined), net household income (OECD), and the Standard International Occupational Prestige Scale based on Helmert et al. [42]. Missing values were imputed using multiple imputation in SPSS 22 (IBM Corp. Armonk, NY) with five imputations, and the mean value as the final data. The SES was categorized into quintiles.

### Statistical analyses

For the descriptive analyses absolute and relative frequencies were calculated for education level, marital status, socio-economic status, self-reported health, and occupational status. Means and standard deviations were calculated for age, total PA (Counts per minute, from here on CPM), and time spent in the active dimensions of the SLOTH model per day in minutes. The descriptive analyses were done separately for men and women, as well as for the two age groups (under and over 70 years). Each result is shown for the total study sample and separately for those participants, who actually perform these activities. Furthermore, the percentage of people engaging in these activities is shown. To test for significant differences between groups, Mann-Whitney-U-tests were conducted since none of the variables were normally distributed. Binary logistic regressions were used to test if total PA and the time spent in the active SLOTH model domains are significantly associated with several health outcomes (self-rated health, overweight, obesity, ADL). For analyses including objective PA data only participants, who wore the accelerometer for at least 20 h on average per day, were taken into account. All statistical analyses were conducted with SPSS 22.0 (IBM Corp. Armonk, NY).

## Results

Table 2 shows descriptive characteristics of the study population. 50.9% of the participants were female and the mean age was 69.9 ± 3.1 years. 82.1% of the participants had lower secondary education, 59.8% belonged to middle class. 58.0% of the female and 82.2% of the male participants were married, and about half of them had a paid occupation or did volunteer work. The majority of participants (78.8%) rated their health as at least good with decreasing percentage in higher age groups. The mean daily total PA shows a difference between men and women and a decline with higher age. Women over the age of 70 years were overall significantly less physically active than those younger than 70 years (1700.4 ± 432.4 CPM vs. 1840.0 ± 466.7 CPM, z = − 2.6, *p* = <.01). The difference in men was also statistically significant, with older men being less physically active than their younger counterparts (1366.5 ± 316.2 CPM vs. 1475.1 ± 352.8 CPM, z = − 2.9, *p* = <.01). Women were significantly more physically active than men (1770.4 ± 454.6 CPM vs. 1426.1 ± 340.5 CPM, z = − 9.4, *p* = <.001).

**Table 2:** Characteristics of the study population

	Women			Men		
	Total (*n* = 465)	< 70 yrs. (*n* = 210)	≥70 yrs. (*n* = 255)	Total (*n* = 448)	< 70 yrs. (*n* = 227)	≥70 yrs. (*n* = 221)
	n (%)			n (%)		
Education						
Lower secondary education	374 (85.0)	162 (81.8)	210 (87.6)	332 (79.0)	151 (70.5)	181 (87.9)
Upper secondary education	56 (12.7)	31 (15.7)	25 (10.4)	77 (18.3)	56 (26.2)	21 (10.2)
No degree	10 (2.2)	5 (2.5)	5 (2.1)	11 (2.6)	7 (3.2)	4 (2.0)
Socioeconomic status						
Lower class	108 (24.6)	43 (21.9)	64 (26.6)	67 (15.9)	32 (15.0)	35 (16.9)
Middle class	264 (60.1)	112 (57.1)	151 (62.7)	250 (59.4)	122 (57.0)	128 (61.8)
Upper class	67 (15.3)	41 (20.9)	26 (10.8)	104 (24.7)	60 (28.0)	44 (21.3)
Marital status						
Married	251 (58.0)	118 (59.9)	133 (56.4)	347 (82.2)	179 (83.3)	168 (81.2)
Divorced	63 (14.5)	35 (17.8)	28 (11.9)	35 (8.3)	19 (8.8)	16 (7.7)
Widowed	99 (22.9)	33 (16.8)	66 (28.0)	20 (4.7)	4 (1.9)	16 (7.7)
Unwed/single	20 (4.6)	11 (5.6)	9 (3.8)	20 (4.7)	13 (6.0)	7 (3.4)
Occupational status						
Volunteer work only	162 (38.3)	76 (40.0)	86 (38.4)	114 (28.6)	58 (28.0)	56 (29.3)
Paid occupation only	37 (8.8)	26 (13.7)	11 (4.9)	67 (16.8)	42 (20.3)	25 (13.1)
Paid occupation + volunteer work	14 (3.3)	11 (5.8)	3 (1.3)	18 (4.5)	10 (4.8)	8 (4.2)
No occupation	201 (47.5)	77 (40.5)	124 (55.4)	199 (50.0)	97 (46.9)	102 (53.4)
Self-reported health status						
Less good or bad	103 (23.6)	39 (19.8)	64 (27.0)	78 (18.6)	37 (17.1)	41 (20.1)
Good	258 (59.2)	117 (59.4)	139 (58.6)	244 (58.1)	126 (58.3)	118 (57.8)
Very good or excellent	75 (17.2)	41 (20.9)	34 (14.4)	98 (23.4)	53 (24.6)	45 (22.0)
	Mean (SD)			Mean (SD)		
Age (years)	70.0 (3.1)	67.0 (1.4)	72.43 (1.7)	69.8 (3.1)	67.2 (1.5)	72.5 (1.9)
Total PA (CPM)	1770.4 (454.6)	1840.0 (466.7)	1700.4 (432.4)	1426.1 (340.5)	1475.1 (352.8)	1366.5 (316.2)

*CPM* Counts per minute

Table 3 reports the average time spent in the active domains of the SLOTH model stratified by age and sex in minutes per day. Disregarding occupation, where no time data is available, most time is spent on home-based activities (women: 118.5 ± 87.8 min/day; men: 80.2 ± 69.4 min/day), followed by active leisure time (women: 35.9 ± 34.0 min/day; men: 41.9 ± 36.8 min/day), and time in active transport (women: 14.4 ± 8.5 min/day; men: 12.5 ± 9.4 min/day). This pattern is consistent in all gender and age groups. The largest difference by age was found for home-based activities in women, where women ≥70 years spent roughly 20 min more time than younger women. There were sex differences in the amount of time spent in the investigated domains. Men spent statistically significantly more time on leisure activities than women (41.9 ± 36.8 vs. 35.9 ± 34.0 min/day, z = − 2.3, *p* = .02). Women allocated statistically significantly more time to active transport (14.4 ± 8.5 vs. 12.5 ± 9.4 min/day, z = − 3.8, *p* = <.01), home-based activities (118.5 ± 87.8 vs. 80.2 ± 69.4 min/day, z = − 7.1, *p* = <.001), and housework (91.2 ± 69.5 vs. 44.1 ± 37.5 min/day, z = − 11.4, *p* = <.001) per day.

**Table 3:** Time allocation to active SLOTH domains in minutes per day by age and sex

	Women			Men		
	Total (*n* = 465)	< 70 yrs. (*n* = 210)	≥70 yrs. (*n* = 255)	Total (*n* = 448)	< 70 yrs. (*n* = 227)	≥70 yrs. (*n* = 221)
	Mean (SD)			Mean (SD)		
**Leisure, only active**						
Total	21.1 (31.4)	22.8 (29.4)	19.6 (33.0)	20.9 (33.4)	24.6 (36.8)	17.2 (29.2)
Participation rate %	58.7	62.9	55.3	50.0	55.9	43.9
Performers only	35.9 (34.0)	36.2 (29.8)	35.5 (37.5)	41.9 (36.8)	43.9 (39.6)	39.2 (32.9)
**Occupation, including volunteer work**						
Total	^a^			^a^		
Participation rate %	56.8	63.3	51.4	55.6	57.3	53.9
Performers only	^a^			^a^		
**Transport, only active**						
Total	11.9 (9.5)	11.3 (8.4)	12.4 (10.2)	10.5 (9.7)	10.6 (10.1)	10.4 (9.3)
Participation rate %	84.6	85.7	80.8	83.9	86.3	81.5
Performers only	14.4 (8.5)	13.2 (7.6)	15.4 (9.1)	12.5 (9.4)	12.2 (9.9)	12.7 (8.8)
**Home-based activities**						
Total	101.4 (91.3)	93.4 (79.8)	108.0 (99.4)	69.5 (70.2)	70.1 (70.5)	68.8 (70.0)
Participation rate %	85.6	86.7	84.7	86.6	88.1	85.1
Performers only	118.5 (87.8)	107.5 (76.1)	127.5 (95.8)	80.2 (69.4)	79.5 (69.9)	80.9 (69.1)
Housework						
Total	76.5 (72.0)	72.2 (63.7)	80.1 (78.1)	35.4 (37.9)	37.4 (37.8)	33.4 (38.0)
Participation rate %	83.9	85.7	82.4	80.4	82.8	77.8
Performers only	91.2 (69.5)	84.2 (60.9)	97.2 (75.7)	44.1 (37.5)	45.1 (37.1)	42.9 (38.1)
Gardening						
Total	24.9 (39.4)	21.1 (32.9)	28.0 (43.8)	34.0 (48.8)	32.7 (49.2)	35.4 (48.6)
Participation rate %	62.2	61.4	62.8	72.3	70.0	74.7
Performers only	40.0 (43.4)	34.4 (36.2)	44.6 (48.1)	47.1 (51.8)	46.7 (52.9)	47.4 (50.9)

^a^Data not available

*SD* Standard deviation

Table 4 shows the average time spent in the active SLOTH domains stratified by occupation status and sex. Regardless of the occupation status and gender, most time is spent on home-based activities (women with no occupation: 118.3 ± 89.3 min/day; with an occupation: 116.5 ± 86.1 min/day; men with no occupation: 83.2 ± 68.3 min/day; with an occupation: 67.7 ± 75.8 min/day). Participants with a paid occupation spent less time on each domain than those without an occupation. The only exception can be observed in women regarding housework, where employed women devoted more time to housework activities than unemployed women (101.5 ± 76.2 min/day vs. 90.0 ± 69.2 min/day). However, none of the differences by occupation status were statistically significant, regardless of sex.

**Table 4:** Time allocation to active SLOTH domains in minutes per day by occupation status and sex

	Women		Men	
	No occupation (*n* = 360)	Occupation^a^ (*n* = 52)	No occupation (*n* = 312)	Occupation^a^ (*n* = 85)
	Mean (SD)		Mean (SD)	
**Leisure, only active**				
Total	23.0 (33.2)	19.8 (27.3)	22.8 (35.7)	19.8 (27.9)
Participation rate %	61.4	67.3	52.6	56.5
Performers only	37.4 (35.4)	29.4 (28.8)	43.3 (39.2)	35.0 (29.0)
**Transport, only active**				
Total	13.1 (9.4)	10.2 (8.3)	11.7 (10.1)	9.3 (8.0)
Participation rate %	89.7	78.9	91.0	87.1
Performers only	14.6 (8.7)	12.9 (7.3)	12.9 (9.9)	10.7 (7.7)
**Home-based activities**				
Total	107.2 (91.8)	105.3 (88.8)	77.1 (69.2)	61.3 (74.8)
Participation rate %	90.6	90.4	92.6	90.6
Performers only	118.3 (89.3)	116.5 (86.1)	83.2 (68.3)	67.7 (75.8)
Housework				
Total	80.3 (71.1)	87.9 (78.9)	39.7 (38.8)	31.8 (37.1)
Participation rate %	89.2	86.5	86.9	81.2
Performers only	90.0 (69.2)	101.5 (76.2)	45.7 (38.1)	39.1 (37.5)
Gardening				
Total	26.9 (40.7)	17.5 (21.7)	37.4 (46.6)	29.6 (60.4)
Participation rate %	64.7	69.2	79.2	67.1
Performers only	41.5 (44.2)	25.2 (22.4)	47.2 (47.7)	44.1 (69.4)

^a^only paid occupation

*SD* Standard deviation

Table 5 presents the results of the binary logistic regressions for the associations of health indicators with time allocation and total PA adjusted for age and sex. The results indicate that more time spent on leisure activities reduces the risk of bad self-rated health (OR: 0.93; 95%-CL: 0.87, 0.99) and limitations in activities of daily living, such as moderate activities (OR: 0.88; 95%-CL: 0.83, 0.94), bending, kneeling, and stooping (OR: 0.93; 95%-CL: 0.89, 0.97) as well as walking more than 1 km (OR: 0.90; 95%-CL: 0.84, 0.96). Participants without an occupation were more likely to rate their health as not good (OR: 0.60; 95%-CL: 0.40, 0.92) compared to those with a post-retirement occupation. More time spent in active transport seemed to lower the risk of having limitations when walking more than 1 km (OR: 0.79; 95%-CL: 0.65, 0.96), being overweight (OR: 0.80; 95%-CL: 0.69, 0.93), or obese (OR: 0.77; 95%-CL: 0.64, 0.92). The results indicated that higher total PA increases the likelihood for a better self-rated health (OR: 0.36; 95%-CL: 0.20, 0.65) and decreases the risk of having limitations regarding moderate activities (OR: 0.55; 95%-CL: 0.34, 0.89), as well as bending, kneeling, and stooping (OR: 0.38; 95%-CL: 0.24, 0.60). The risk of being overweight (OR: 0.39; 95%-CL: 0.25, 0.62) or obese (OR: 0.36; 95%-CL: 0.21, 0.60) also decreased with increased total PA. Time spent in home-based activities did not seem to be associated with any of the health outcomes.

**Table 5:** Results of binary logistic regression on predicting health indicators, adjusted for age and sex

	Bad self-rated health	Limitations when doing moderate activities (ADL)	Limitations when bending, kneeling, stooping (ADL)	Limitations when walking > 1 km (ADL)	Overweight	Obesity
	OR (95%-CL)	OR (95%-CL)	OR (95%-CL)	OR (95%-CL)	OR (95%-CL)	OR (95%-CL)
Leisure, only active (hrs/week)	**0.93 (0.87, 0.99)**	**0.88 (0.83, 0.94)**	**0.93 (0.89, 0.97)**	**0.90 (0.84, 0.96)**	0.96 (0.92, 1.00)	0.96 (0.91, 1.01)
Occupation (yes/no)	**0.60 (0.40, 0.92)**	0.90 (0.63, 1.30)	0.90 (0.65, 1.25)	0.77 (0.52, 1.13)	0.76 (0.53, 1.08)	0.80 (0.54, 1.17)
Transport, only active (hrs/week)	0.96 (0.79, 1.16)	1.07 (0.91, 1.25)	0.94 (0.81, 1.09)	**0.79 (0.65, 0.96)**	**0.80 (0.69, 0.93)**	**0.77 (0.64, 0.92)**
Home-based activities (hrs/week)	0.99 (0.97, 1.01)	0.99 (0.98, 1.01)	1.00 (0.99, 1.02)	0.99 (0.97, 1.01)	0.99 (0.97, 1.00)	0.98 (0.96, 1.00)
Total PA^a^ (CPM)	**0.36 (0.20, 0.65)**	**0.55 (0.34, 0.89)**	**0.38 (0.24, 0.60)**	0.61 (0.36, 1.02)	**0.39 (0.25, 0.62)**	**0.36 (0.21, 0.60)**

^a^Only participants with at least 20 h/day of accelerometer data included (*n* = 570)

*ADL* Activities of daily living, *CL* Confidence limits, *CPM* Counts per minute, *OR* Odds ratio

Statistically significant results in bold

## Discussion

This study investigated age and sex specific differences in time use in three active domains (leisure, transport, home-based) and examined associations between time allocation in these domains and objectively measured PA with different health outcomes (self-rated health, BMI, ADL). Furthermore, we explored differences in time allocation between working and non-working older adults. Results showed age and sex differences in total PA as well as in time use in the active domains. Participants with a paid occupation spent less time in almost every active domain than participants without an occupation. Furthermore, the results showed several associations between active domains and health indicators. While total PA was associated with all investigated health indicators, time spent in the different SLOTH domains showed a more distinctive pattern, with leisure activities and active transport being most influential. Time spent in active leisure time was associated with better self-rated health and having no limitations in ADL. Time spent on active transport was associated with having no limitations when walking more than 1 kilometer and being normal weight. Having an occupation was only associated with better self-rated health. Home-based activities showed no significant associations with any of the investigated health indicators.

Our results regarding total PA are in line with previous research. Total PA is linked to better self-rated health [43, 44], overweight and obesity [45], and physical functioning [46, 47]. Our study showed a statically significant higher objectively measured PA in women compared to men. These results are in contrast to several other studies objectively measuring PA in older adults. The systematic review by Sun et al. [3], for instance, found older men to be more physically active than older women. These differences could arise from differences in PA measurements. In this study the accelerometer was worn on the non-dominant wrist, since it tends to have a higher compliance compared to hip placement [48]. However, most studies use PA monitors on the hip, which results in differences in the measured movements, especially regarding the upper body [3]. Since the women in our sample devote a lot of time to housework, which includes lots of upper body movements, it is possible that the higher amount of PA stems from this. Moreover, our study showed that participants older than 70 years were significantly less physically active than those under 70 years. Yet, they spent more time in several active SLOTH domains with increasing age. One reason could be the decreasing proportion of people having a paid occupation or doing volunteer work as they get older (women: 63.3% compared to 51.4%, men: 57.3% compared to 53.9%), which leads them to allocate their time differently. Another possibility is that with increasing age, more time might be required to perform certain tasks, because of the ageing-related decline in health [49]. Both men and women in the older age group (age 70+ years) more often reported less good or bad health than the younger ones. Since our data only provides information about the time allocation to active domains and cannot provide information about the intensity, we can only speculate that although the time spent in the SLOTH domains increases with higher age, the intensity might decrease. Spinney et al. [50], for example, found decreasing rates of older Canadians meeting PA recommendations with increasing age, when looking at the active domains.

In our study, women reported spending 35.9 and men 41.9 min per day in active leisure. In line with our results, a systematic review [24] reported average leisure time of 0.5 to 1 h per day, with men devoting more time to it than women. Krantz-Kent and Stewart [51] found similar results in their American study. Our findings further indicated that time spent in active leisure is associated with better self-rated health, which is supported by the studies by Abu-Omar and Rotten [11], as well as Kaleta and colleagues [52]. Both of these studies, however, did not focus on older adults specifically. Time doing leisure activities was also associated with lower risks of having limitations in ADL, which is in accordance with previous research [16, 53]. The results emphasize the importance of active leisure for health.

Since we did not assess time spent on occupational PA, we have compared the time spent in the remaining domains between employed and unemployed participants. The results indicated that women and men without an occupation spent more time in each domain than the participants with an occupation. The only exception is working women, who spent more time doing housework than their non-working counterparts. A study by Flood and Moen [54] also found that employment is negatively associated with time being physically active. Having an occupation was associated to better self-rated health in our study. Research on health effects of occupation in older adults is scarce, as often is the focus rather on the effects of retirement. A systematic review on retirement found contrasting results in the literature, with retirement having positive as well as adverse effects on health [19]. One possible explanation are diverging definitions of occupation and retirement, for example if doing unpaid volunteer work counts as having an occupation.

Our participants only spent a short time (Women: 14.4 min/day; Men: 12.5 min/day) in active transport. Sprod and colleagues [23, 55] reported almost three times more minutes per day being spent on active travel. These differences could be a result of us only using everyday destinations, whereas Sprod et al. included every destination. Another explanation could be the difference in infrastructure between the study countries (Australia vs. Germany), that leads to the participants having to cover longer routes. Time spent in active transport was associated with a lower risk of overweight and obesity. Additionally, it was associated with a lower risk of having limitations in walking more than 1 km. In this context, limited mobility could be a barrier to PA and using active transport. We therefore performed a sensitivity analysis excluding participants who rely on mobility aids. The results do not change by much although they are statistically significant anymore (before: OR: 0.79, 95%-CI: 0.65, 0.96; sensitivity analysis: OR: 0.86, 95%-CI: 0.70, 1.05). Furthermore, possible reverse causation should be taken into account, since the data only stems from a cross-sectional study. A comparison with existing research is difficult, since other time use studies often do not distinguish between active and inactive transport or define it as total PA [54] or commuting [11]. A study by Foley et al. [20] could, however, associate time in active transport with spending more time on healthy behaviours. In summary, participation in active transport offers a good opportunity for older adults as health promoting behaviour.

Our results show sex differences in time allocation regarding home-based activities. The sex differences are in line with previous research [12, 23, 55], with women spending more time on household chores than men. Gauthier and Smeeding [24] reported the same findings for overall home-based activities in nine different countries, but, contrasting our results, saw a decrease with age in women devoting time to housework and an age-related increase in men. We found no associations of home-based activities and health outcomes, which is supported by previous research, supporting the lack of associations of household chores and overweight [22] or health status [21]. Contrastingly, Adjei and Brand [12] found that older adults who spent more time doing home-based activities are more likely to report good health. These differences could stem from different measurements of home-based activities, including time-use diaries for one, 2 or 7 days with 24 h of data [12].

The study has a few limitations that need to be addressed. The questionnaire used in the OUTDOOR ACTIVE study was not initially designed for time use analyses, thus some domains of the SLOTH model were not fully assessed. Furthermore, we have not performed any type of reproducibility of the questionnaire. However, several components from validated questionnaires were used in our study [38–40]. Moreover, the assessment of housework and gardening might be biased, since it was not clarified which tasks account to these domains. It is, for example, unknown whether the participants included cooking to housework or only referred to cleaning. Moreover, the activity “riding a bike” was excluded from the leisure domain, since it is possible that participants included time spent on riding a bike for transport in their answer. This could lead to underestimated time in this domain. Since the participants had to estimate their weekly time spent in leisure and household activities, recall bias and reporting errors could be an issue. The use of a proper time use diary could reduce these risks. Additionally, they would deliver data for full 24-h days. However, the results of the present study are still a good indicator for the time allocation to active domains in older adults. The assessment of transport only included everyday destinations, leaving out travelling to work, social events, or other obligations, which could lead to an underestimation of time allocation. Another limitation of this study is the cross-sectional design. Therefore, no statements regarding causation can be made and associations because of possible reverse causation (e.g. between PA and self-rated health, between occupation and self-rated health, or between active transport and limitations when walking more than 1 km) cannot be determined. Thus, future research should look at time allocation and PA, and its effect on health outcomes in a longitudinal study.

One strength of this study is the representativeness of the sample for the population of Bremen-Hemelingen, when comparing social demographic factors. Furthermore, the PA data was assessed objectively using accelerometers, which is a reliable measurement for PA in older adults [6]. Furthermore, the SLOTH model is a well-known and fitting time budget model to analyse time allocation regarding PA [56].

This study provided insights in time allocation of older adults to active domains and PA, which are important information for developing PA programs and guidelines. Additionally, associations of time use and several health indicators were presented. These findings can help to develop new PA guidelines, which mostly incorporate overall PA in minutes and intensity but do not take the different life domains into consideration. If guidelines also include domains, specific PA promotion programs could be designed accordingly. Additionally, the knowledge of the associations between health indicators and PA in different domains can help practitioners in tackling health problems. For example, overweight and obesity prevention programs could incorporate the promotion of active transport, which might be easier for some participants to realise than starting and following an exercise programme.

## Conclusions

The findings of this study have shown differences by age, sex, and occupational status in time allocation to active domains of daily living as well as in objectively measured PA in older adults. Furthermore, time use was associated with several health outcomes. Healthy ageing, and in this context also active ageing, is an important public health goal. This study provides information on how older adults structure their day, which active domain they devote most time to, and which health indicators are associated with active domains, which has important implications for the development of PA programs. It shows that PA is beneficial for healthy ageing and that active domains should be integrated in PA promotion and the development of PA guidelines. For example, active transport could be used for obesity prevention, and the increase of leisure activities for reducing limitations in ADL. However, further research and longitudinal studies are needed to understand the specific links between time allocation to active domains and health indicators to increase active living as a part of healthy ageing.

## Supplementary information

**Additional file 1.** Estimated number of visits per week to common destinations. To calculate time in active transport, we estimated the number of visits per week to the common destinations, that were used in the questionnaire.

## Data Availability

The datasets used and/or analysed during the current study are available from the corresponding author on reasonable request.

## Abbreviations

ADL: Activities of Daily Living
BMI: Body Mass Index
CPM: Counts per minute
MVPA: Moderate to vigorous physical activity
PA: Physical activity
SES: Socioeconomic status
SLOTH model: Sleep, Leisure activities, Occupation, Transport, Home-based activities
