# Supplementary material for: Time allocation to active domains, physical activity, and health indicators in older adults: cross-sectional results from the OUTDOOR ACTIVE study
Source: BMC Public Health. 2020 Oct 20;20:1580. doi: 10.1186/s12889-020-09708-z (PMC7576691; doi:10.1186/s12889-020-09708-z)
Supplement: Supplementary file 1 — Additional file 1. Estimated number of visits per week to common destinations. To calculate time in active transport, we estimated the number of visits per week to the common destinations, that were used in the questionnaire. [file 12889_2020_9708_MOESM1_ESM.docx]

**Additional files**

**Additional file 1** Estimated number of visits per week to common destinations

| **Destination** | **Estimated number of visits per week** |
| --- | --- |
| Supermarket | 4 |
| Bakery | 4 |
| Café | 2 |
| Restaurant | 0.5 |
| Post office | 1 |
| Bank | 1 |
| Hair dresser | 0.5 |
| General practitioner | 0.5 |
| Pharmacy | 0.5 |
| Cemetery | 1 |
| Bus stop | 4 |
| Sports facility | 2 |
